# Changes of Atmospheric and Blood Concentrations of Lead and Cadmium in the General Population of South Korea from 2008 to 2017

**DOI:** 10.3390/ijerph16122096

**Published:** 2019-06-13

**Authors:** Jaeouk Ahn, Nam-Soo Kim, Byung-Kook Lee, Inbo Oh, Yangho Kim

**Affiliations:** 1 Department of Medical IT Engineering, Soonchunhyang University, 22 Suncheonhyang-ro, Asan 31538, Korea; jaeouk.ahn@gmail.com; 2 Institute of Occupational and Environmental Medicine, Soonchunhyang University, 22 Soonchunhyang-ro, Asan 31538, Korea; kns0903@sch.ac.kr; 3 Department of Preventive Medicine, Soonchunhyang University, 22 Suncheonhyang-ro, Asan 31538, Korea; bklee@sch.ac.kr; 4 Environmental Health Center, Ulsan University Hospital, University of Ulsan College of Medicine, 877 Bangeojinsunhwando-ro, Dong-gu, Ulsan 44033, Korea; oinbo@naver.com; 5 Department of Occupational and Environmental Medicine, Ulsan University Hospital, University of Ulsan College of Medicine, 877 Bangeojinsunhwando-ro, Dong-gu, Ulsan 44033, Korea

**Keywords:** lead, cadmium, air concentration, blood, exposure

## Abstract

We measured changes in atmospheric and blood levels of lead and cadmium in the South Korean general population during the past decade. Blood data of 16,873 adults were taken from the Korean National Health and Nutrition Examination Survey (KNHANES, 2008–2017). Atmospheric data were taken from 66 air quality monitoring sites in 16 different regions in South Korea. From 2008 to 2017, the geometric mean (GM) blood lead level decreased from 2.37 to 1.46 μg/dL (38.4% decrease), and the atmospheric lead concentration decreased by 61.0% in the overall population. During this time, the GM blood cadmium level decreased from 0.88 to 0.72 μg/L (18.2% decrease), and the atmospheric cadmium concentration decreased by 63.6%. Multiple linear regression analysis indicated that a half reduction in air lead was associated with a 0.09 μg/dL decrease in blood lead (95% CI: 0.03 to 0.15) in a subgroup of the metropolitan city population. However, a half reduction in air cadmium had no significant effect on blood cadmium. Multiple linear regression analyses indicated that the decrease in blood lead level over 10 years in Korea was related to the decrease in atmospheric lead concentration. However, the decrease in blood cadmium level during this time was not significantly associated with the decrease in atmospheric cadmium concentration. Our findings suggest that inhalation is a major source of lead exposure, but not of cadmium exposure. Ingestion of dietary cadmium presumably has a stronger impact on blood cadmium levels.

## 1. Introduction

Lead and cadmium are widely dispersed in the environment, and there is increasing evidence that the general populations of many regions may be exposed to harmful levels of these heavy metals. Industrial workers may be exposed to these metals at their places of employment, but general populations are mainly exposed via non-occupational sources [1,2]. 

Lead can damage the central nervous system, kidneys, cardiovascular system, reproductive organs, and hematological system. A recent paper reported that blood lead levels in children that were even well below the 10 μg/dL were associated with reduced IQ, deficits in executive function, and attention deficit hyperactivity disorder (ADHD) [3]. For adults, exposure to low levels of lead can reduce kidney function, increase blood pressure, increase the risk of miscarriage in women, and reduce sperm production in men [4]. The main environmental sources of lead are leaded gasoline, lead paint (including lead paint-contaminated dust and soil), water from lead pipes, and industrial emissions [1,5]. Cigarette smoking and consumption of certain foods are also sources of lead exposure [1,6]. Thus, environmental lead can enter the body by inhalation or ingestion [7]. Blood lead concentration accounts for part of the total body burden, because it reflects uptake from recent exposure and slow release from the endogenous skeletal pool [8]. Lead has a short half-life in blood (20 days) and soft tissue (40 days), and a long half-life in bone (10 to 30 years). Hence, a person’s blood lead level reflects recent exposure [9].

Cumulative exposure to cadmium can increase the risk for cardiovascular, neurological, renal, and developmental diseases [2]. Recent research showed that prenatal exposure to a low level of cadmium had adverse effects on neurodevelopment [10,11]. Environmental cadmium is ubiquitous in air, soil, and water due to industrial activities, use of phosphate fertilizers, combustion of motor fuels, and particles released by tire wear [2,12,13]. Cadmium levels in the body increase with age, as only a minute part of the body burden (0.01–0.02%) is excreted each day [2]. Among nonsmokers, diet is the major source of cadmium exposure; among smokers, tobacco is the major source because tobacco, like most other plants, absorb cadmium [14]. The level of blood cadmium—which has a half-life of 3–4 months [15]—is considered the most reliable biomarker of recent exposure [15,16], although the level of urinary cadmium—which has a half-life of 15–30 years—is a reliable biomarker of lifetime exposure [17]. After long-term cadmium exposure, an increasing proportion of blood cadmium will account for the total body burden [16,18].

Previous research has documented reductions of atmospheric and blood levels of lead following the prohibition of leaded fuels. For example, in the U.S., after the prohibition of lead-based paint (from 1973 to 1995) and leaded fuels from cars, airplanes, leisure vehicles, and ships (in 1996) [19], the number of individuals with blood lead levels of 10 μg/dL or more decreased from more than 10 per 1000 (in late 1990s) to about 6 per 1000 (in 2009) [20]. In Taiwan, unleaded fuels were introduced in 1986, and leaded fuels were prohibited in 2000; this resulted in a decrease of both atmospheric lead concentration and blood lead levels from 1985 to 2002 [21]. However, few studies have examined changes over time in atmospheric and blood levels of lead and cadmium in general populations [22,23]. 

The present study evaluated changes in the atmospheric and blood levels of lead and cadmium from 2008 to 2017 in the general population of South Korea.

## 2. Materials and Methods 

### 2.1. Design and Data Collection

This cross-sectional study used data from the Korea National Health and Nutrition Examination Survey (KNHANES) of 2008–2017, which included KNHANES IV (2007–2009), KNHANES V (2010–2012), KNHANES VI (2013–2015), and KNHANES VII (2016–2017). These surveys were conducted annually by use of a rolling sample design that employs a complex, stratified, multistage probability cluster analysis of a representative sample of the non-institutionalized civilian population of South Korea. Thus, the KNHANES is a large representative population study with rigorous quality controls. These surveys were performed by the Korean Centers for Disease Control and Prevention and the Korean Ministry of Health and Welfare, and have three components: a health interview, a health examination, and a nutrition survey. 

After explanation of the survey, all participants were provided written informed consent prior to participation. This survey was approved by the Institutional Review Board of the Korean Centers for Disease Control and Prevention (approval nos. 2007-02CON-04-P, 2008-04EXP-01-C, 2009-01CON-03-2C, 2010-02CON-21-C, 2011-02CON-06-C, 2012-01EXP-01-2C, 2013-07CON-03-4C, 2013-12EXP-03-5C). 

The analysis was restricted to 16,873 adults (≥19 years old) who completed the health examination survey, which included measurement of blood metals, and the nutrition survey. Kweon provided details on the survey design [24]. Briefly, data on age, sex, smoking history, alcohol intake, region of residence, and occupation were collected during the health interview. Smoking status (current smoker, past smoker, and never-smoker) was based on self-reported cigarette use. Never-smokers were those who had smoked fewer than 100 cigarettes in their lifetimes; subjects who smoked 100 or more cigarettes were classified as past or current smokers based on current smoking habits. Alcohol consumption was based on self-reported drinking behavior during the month prior to the interview, and included average frequency of drinking (days per month) and amount ingested (in mL) on each occasion. These data were converted into amount of pure alcohol (g) consumed per day. Subjects were then categorized according to average daily alcohol consumption: nondrinker, light drinker (1–15 g), moderate drinker (16–30 g), or heavy drinker (>30 g). Area of residence was categorized as urban (within an administrative division of a city) or rural (outside the administrative division of a city). Regular walking was defined as indoor or outdoor walking for at least 30 min at a time at least 3 times per week. Regular exercise was defined as participating in moderate exercise (slow swimming, doubles tennis, volleyball, or occupational or recreational activity involving the carrying of light objects) on a regular basis for at least 30 min at a time at least 5 times per week, or participating in vigorous exercise (running, climbing, fast cycling, fast swimming, football, basketball, rope jumping, squash, singles tennis, or occupational or recreational activity involving the carrying of heavy objects) for at least 20 min at a time at least 3 times per week. Study subjects were classified as waged employees or others (self-employed, employers, or unemployed), and waged employees were further categorized as manual or non-manual workers.

### 2.2. Determination of Lead and Cadmium in Whole Blood

To assess the levels of heavy metals in whole blood, 3 mL samples were drawn into standard commercial evacuated tubes containing sodium heparin (Vacutainer®; Becton, Dickinson and Company, Franklin Lakes, NJ, U.S.). Lead and cadmium were measured using graphite furnace atomic absorption spectrometry with Zeeman background correction (Analyst™ 600; Perkin Elmer, Turku, Finland). All blood metal analyses were performed by the Neodin Medical Institute, a laboratory certified by the Korean Ministry of Health and Welfare. Analytic analyses and quality control are described in a previous paper [25].

### 2.3. Environmental Heavy Metal Measurements

To assess the average atmospheric concentrations of lead and cadmium in 16 different regions, including 7 metropolitan cities in South Korea, we obtained daily measurements over 10 years (2008–2017) from 66 nationwide air quality monitoring sites operated by the Korea Ministry of Environment. Annual average geometric mean (GM) concentrations were calculated based on monthly validated daily data.

### 2.4. Statistical Analysis

Statistical analyses were performed using SAS software (ver. 9.4; SAS Institute, Cary, NC, USA) and SUDAAN (Release 11.0; Research Triangle Institute, Research Triangle Park, NC, USA), a software package that incorporates sample weights and adjusts for complex sample design. Survey sample weights were used in all analyses to produce population estimates that were representative of the non-institutionalized civilian population of Korea.

The blood levels of each heavy metal were log-transformed because their distributions were positively skewed. The adjusted GM and 95% confidence interval (CI) were calculated according to sex, year of study, age group, urban or rural residence, employment status, physical activities (exercising or walking), smoking status, and drinking status using the Proc Regress function in SUDAAN. We compared blood lead or cadmium concentrations in post-menopausal women and pre-menopausal women in the 50–59 cohorts. We limited our analysis to 50–59 cohorts because of the increase in blood lead and cadmium concentration with age. 

Multiple regression analyses with the standard method of entry were performed to determine the relationship of blood levels of heavy metals with environmental air concentrations of heavy metals in individuals of seven metropolitan cities (*n* = 7889) in a gender-specific way. Each of these seven cities has many air monitoring sites that provide reliable data. Other regions have fewer air monitoring sites, and thus their data on air concentrations of heavy metals are less reliable to represent exposure concentration of individuals living in the region. Hence, we used air metal concentrations in the monitoring sites limited to the population of the metropolitan cities to assess an association of air concentrations with blood metal concentration. The beta coefficient (95% CI) of blood metal levels of this subgroup was calculated using air concentrations as an independent variable, after adjustment for covariates (age, sex, urban or rural residence, region of residence in Korea, physical activities, employment status, smoking, and drinking status). 

## 3. Results

Table 1 shows the general demographic characteristics of the study subjects. There were no gender differences in age distribution, area of residence, or exercise, but there were gender differences in employment status, smoking, alcohol consumption, and performance of regular walks. 

### 3.1. Changes in Blood Lead and Atmospheric Lead Over Time

Table 2 shows the blood lead levels of adults overall and of different subgroups during each year. Overall, the GM blood lead level decreased from 2.37 μg/dL in 2008 to 1.46 μg/dL in 2017 (a 38.4% decrease). Men had higher lead levels than women during each year, but the annual decreases were similar in the two genders. Old individuals had higher levels than young individuals during each year, and annual declines were greater in those over 60 years than in those younger than 40 years. Manual workers had higher levels than the other employment groups, and the annual changes were greater in manual and non-manual employees than others. Current smokers had higher levels than non-smokers and past smokers, but the three groups had similar annual declines. Heavy drinker had higher level values when compared to than non-drinkers, but the annual declines were smaller for non-drinkers. Residents in rural areas had higher levels than those in urban areas, but those in urban areas had greater annual declines. Individuals who did and did not engage in outdoor activities (such as exercise or regular walking) had no differences in blood lead concentration, and similar annual declines. Menopausal women had higher levels than pre-menopausal women, and the annual declines were smaller in menopausal women.

**Figure 1:**
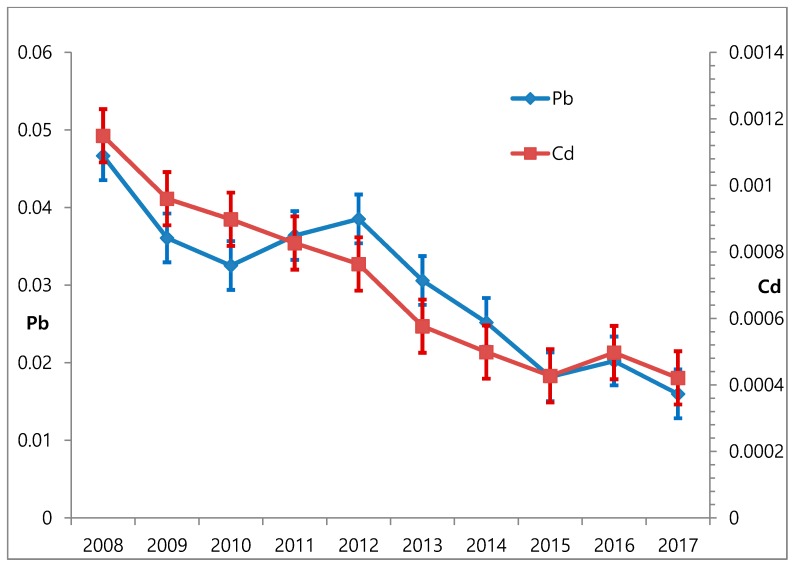
Changes of atmospheric GM concentrations of lead (Pb) and cadmium (Cd) from 2008 to 2017.

Figure 1 shows the annual atmospheric GM lead concentrations of 66 nationwide air quality monitoring sites from 2008 to 2017. The GM concentration was 0.0467 μg/m^3^ in 2008 and 0.0182 μg/m^3^ in 2017. Thus, from 2008 to 2017, atmospheric lead concentration decreased by 61.0% and blood lead concentration decreased by 38.4%.

### 3.2. Changes in Blood Cadmium and Atmospheric Cadmium over Time

Table 3 shows the annual GMs of blood cadmium of adults overall and of different subgroups. Overall, the blood cadmium level decreased from 0.88 μg/L in 2008 to 0.72 μg/L in 2017 (a 18.2% decrease). In general, the declines of blood cadmium were not as strong or as consistent as those of blood lead. Women had higher levels than men, but the annual decreases were similar in men and women. Older individuals had much higher levels than young individuals, and the annual declines were greater in those over 40 years than in those younger than 40 years. Non-manual workers had lower levels than those in other employment groups, and the annual declines were similar for all three groups. Current smokers had higher levels than non-smokers and past smokers, and the annual declines were greater in non-smokers than smokers. Heavy drinkers had higher levels than non-drinkers, however, the annual decline was smaller in non-drinkers than others. Residents of rural areas and urban areas had similar levels, and the annual decline was similar in these two groups. Individuals who performed outdoor activities had lower levels than those who did not. The annual decrease was greater in those who performed exercise than those who did not; however, the decline was greater in those who did not perform regular walks than those who did. Menopausal women had lower levels than pre-menopausal women, and the annual declines were greater in menopausal women.

Figure 1 shows the annual atmospheric GM cadmium concentrations from 2008 to 2017 in 66 nationwide air quality monitoring sites. The average concentration was 0.0011 μg/m^3^ in 2008 and 0.0004 μg/m^3^ in 2017. Thus, from 2008 to 2017, the atmospheric cadmium concentration decreased by 63.6%, and the blood cadmium concentration decreased by 18.2%.

### 3.3. Relationship of Atmospheric and Blood Levels of Lead and Cadmium in Men and Women

We also estimated the adjusted mean difference of blood lead and cadmium concentration associated with doubling of the air concentration of each heavy metal in men and women (Table 4). In this analysis, we regressed blood concentrations against log_2_-transformed air concentrations for the 7899 individuals who lived in 7 metropolitan cities after adjustment for covariates (gender, age, residence areas, region of residence in Korea, employment status, smoking and drinking status, and performance of outdoor activities). The multiple linear regression results indicated that a doubling of atmospheric lead was associated with an increase of 0.09 (95% CI: 0.03 to 0.15) and 0.10 μg/dL (95% CI: 0.03 to 0.17) of blood lead in the overall population and women, respectively. Thus, a half reduction in air lead was associated with a decrease of 0.09–0.10 μg/dL. Moreover, a decrease in blood lead concentration associated with gender (female vs. male) was 0.40 μg/dL. The increase in blood lead concentration associated with age (30–39, 40–49, 50–59, and ≥60 vs. age younger than 30) was 0.27, 0.53, 0.82, and 0.90 μg/dL, respectively, indicating a graded increase with age. Both genders showed a similar increasing trend with age; however, the increases were greater in men than women. The increase in blood lead concentration associated with smoking (current smoker vs. non-smoker) was 0.18 μg/dL and 0.17 μg/dL in the overall population and men, respectively. The increase in blood lead concentration associated with alcoholic consumption (mild, moderate, and heavy alcoholic consumption vs. no consumption) was 0.09, 0.39, and 0.52 μg/dL, respectively, indicating graded increase with alcoholic consumption. Both genders showed a similar increasing trend.

Application of the same analysis for cadmium showed no significant effect in the overall population (Table 4). However, the increase in blood cadmium concentration associated with gender (female vs. male) was 0.31 μg/L. The increase in blood cadmium concentration associated with age (30–39, 40–49, 50–59, and ≥60 vs. age younger than 30) was 0.20, 0.48, 0.60, and 0.69 μg/L, respectively, indicating graded increase with age. Both genders showed a similar increasing trend with age; however, the increases were higher in women than men. The increase in blood cadmium concentration associated with smoking (current smokers vs. non-smoker) was 0.48, 0.25, and 0.39 μg/L in men, women, and the overall population, respectively. Male past smokers had higher cadmium concentration than non-smokers. The increase in blood cadmium concentration associated with alcoholic consumption (heavy alcoholic consumption vs. no consumption) was 0.11, 0.14, and 0.11 μg/L in men, women, and the overall population, respectively.

## 4. Discussion

This research described the annual changes in the atmospheric and blood GMs of lead and cadmium in the general Korean population during that past decade. Although people can be exposed to lead through various sources, inhalation is the most common source if atmospheric lead concentration is high [1]. Our multiple linear regression analysis found that the decrease in blood lead level was associated with the decrease in atmospheric lead level. Furthermore, annual decline in blood lead was consistent irrespective of several parameters related to blood lead levels, although minor differences in annual decline were shown according to the parameters. These findings suggest that inhalation is the major source of lead exposure in the Korean general population. 

Several air pollution research projects in the U.S. and Europe has documented the relationship between lead exposure from air and soil and child blood lead levels [22,23]. Other research has documented a decline in atmospheric lead concentration and blood lead levels following the regulation of leaded fuels [26,27]. In Korea, the drastic decrease in annual atmospheric lead concentration from 1989 to 2000 may be attributable to the regulation of leaded fuels and the imperative for use of non-leaded fuels [28]. The results of Park support this observation [29]. Although the annual average atmospheric lead concentration peaked in 1989 and decreased thereafter, the blood lead levels of Korean people peaked in 1992 and then decreased. This decrease in blood lead concentration could be attributed to the successful regulation of leaded fuels [30]. Furthermore, the present study showed there was a persistent decrease in the air lead level from 2008 to 2017. The current sources of atmospheric lead are emissions from coal combustion and industrial activities [31], rather than leaded fuels. 

The present study also found that the decrease in blood cadmium was smaller than that of blood lead, although there were similar declines in the atmospheric levels of lead (61.0%) and cadmium (63.6%). Furthermore, our multiple linear regression analysis indicated no significant association of blood cadmium and atmospheric cadmium concentrations. These findings suggest that ingestion (not inhalation) is the main source of cadmium exposure in the general Korean population. In agreement, Moon et al. [32] studied the general population of South Korea and found that diet was the main source of cadmium exposure [33]. Several other studies also found that blood cadmium levels were higher in individuals from northeastern Asia (Korea, Japan, and China) than in those from Western countries, possibly because of lower rice consumption in the West [34,35]. Fish has also been shown to be a possible source, although not a major source, of cadmium [36].

In our sample of the Korean general adult population, the GM levels of blood lead (1.46 μg/dL) and blood cadmium (0.72 μg/L) during 2017 were higher than reported in the recent US NHANES for adults (lead: 0.920 μg/dL, cadmium: 0.295 μg/L) [37]. This could be due to differences in diet, environmental exposures, or sampling and analytic methods [32,35]. In U.S., as a result of EPA’s regulatory efforts including the removal of lead from motor vehicle gasoline, levels of lead in the air decreased by 99 percent between 1980 (1.948 μg/m^3^) and 2017 (0.015 μg/m^3^) [38]. Current lead concentration in U.S. is similar to 0.018 μg/m^3^ in the present study. Urban background atmospheric cadmium concentrations (including traffic related sites) were 0.2–2.5 ng/m^3^ in Europe, which are comparable to 0.4 ng/m^3^ in the present study [39].

The present study showed gender differences in the behavior of blood metals; for example, males had higher blood concentrations of lead than females, but females had higher blood concentrations of cadmium than males. Previous studies also reported sex differences in blood lead and cadmium levels in adults [25,40,41]. The higher cadmium concentration in female adults is partly due to iron deficiency associated with menstruation. In particular, the divalent metal transporter 1 (DMT1) mediates the uptake of cadmium and iron, and its expression is upregulated in the presence of low iron stores. Thus, there is an increased cadmium uptake and blood cadmium concentration in the presence of iron depletion [41,42,43,44]. In agreement, menopausal women had lower cadmium levels than pre-menopausal women in the present study and a previous study [45]. After menopause, when there is no more monthly loss of iron, the increased iron stores result in lower absorption of dietary cadmium and lower blood cadmium concentrations [45]. However, menopausal women had higher lead levels than pre-menopausal women in the present study. After menopause, when lead may be mobilized from the skeleton due to increased bone demineralization, blood lead concentrations increase compared to the pre-menopausal period [45,46]. An increase in blood lead concentrations with age was more significant in men, whereas the rise in blood cadmium concentrations with age was higher in women. The increase in blood cadmium concentration associated with smoking status was more significant in men.

Advanced age is a significant risk factor for higher levels of heavy metals because of bioaccumulation. In fact, we observed that the levels of blood lead and especially cadmium increased with age. Our previous report [47] showed that the concentrations of blood lead and cadmium were clearly greater in older individuals. In addition, we found that blood lead levels were higher in individuals from rural areas than in those from urban areas. In general, urban environments are more polluted than rural areas [48,49]; however, factories are increasingly being built in rural areas of Korea and Koreans living in urban areas tend to have higher socioeconomic status. Physical activities (walking and exercise) may cause inter-individual variations in the uptake of air contaminants, and thereby affect blood metal concentrations [50]. We found an association of exercise with blood cadmium, but no such association for blood lead. In addition, we also found higher levels of blood lead and cadmium in smokers than nonsmokers [14,25]. Manual workers had higher blood lead and cadmium levels than non-manual workers, possibly because of occupational exposures.

The present findings have important public health implications. The modifiable determinants of blood heavy metal levels are the ambient atmosphere (lead) and diet (cadmium). Thus, public health policy should implement different strategies to reduce exposures to each heavy metal. These strategies should monitor blood levels and evaluate exposure route and the contribution of different sources in an effort to reduce exposures.

This study has several important strengths. First, we used a representative sample of the general population of South Korean adults. Second, the KNHANES employed rigorous quality control of study procedures. However, this study also had some limitations. First, we determined all associations by cross-sectional analysis, as in many previous reports. Therefore, we cannot infer causality for any of these associations, because unknown intermediating factor(s) might explain the observed associations. A prospective study will be required to establish a causal basis for these associations.

## 5. Conclusions

The results of our multiple linear regression analysis of the Korean general population indicated that the decrease in blood lead concentration was related to the decrease in atmospheric lead concentration. This suggests that inhalation is a major source of lead exposure. However, the multiple linear regression results indicated that the decline in atmospheric cadmium concentration was not responsible for the decline in blood cadmium concentration. This suggests that main source of cadmium exposure is ingestion rather than inhalation.

## Figures and Tables

**Table 1:** General demographic characteristics of study subjects.

Classification		Total	Men	Women
		(*n* = 16,873)	(*n* = 8117)	(*n* = 8756)
Age, NS	<30	3011 (25.2)	1451 (26.4)	1560 (23.6)
	30–39	3258 (20.1)	1570 (20.6)	1688 (19.5)
	40–49	3341 (21.4)	1621 (21.4)	1720 (21.3)
	50–59	3376 (18.8)	1612 (18.7)	1764 (18.9)
	60≤–	3887 (14.6)	1863 (12.9)	2024 (16.8)
Employment*	Manual	4102 (26.4)	2319 (29.7)	1783 (22.4)
	Non-manual	6397 (38.2)	3722 (45.2)	2675 (29.7)
	Others	6374 (35.4)	2076 (25.1)	4298 (47.9)
Smoking*	Non-smoker	9461 (52)	1758 (23.8)	7703 (86.4)
	Past smoker	3344 (19.7)	2853 (30.7)	491 (6.2)
	Current smoker	4068 (28.3)	3506 (45.4)	562 (7.4)
Alcohol*	None	4274 (21.8)	1302 (14.3)	2972 (30.9)
	Mild	8414 (50.5)	3493 (44.7)	4921 (57.6)
	Moderate	2004 (12.6)	1442 (17.1)	562 (7.2)
	Heavy	2181 (15.1)	1880 (24)	301 (4.3)
Residence, NS	Urban	13671 (83.4)	6544 (83)	7127 (83.9)
	Rural	3202 (16.6)	1573 (17)	1629 (16.1)
Outdoor activity	Exercise, NS	2923 (21.5)	1395 (21.3)	1528 (21.9)
	No exercise	13950 (78.5)	6722 (78.7)	7228 (78.1)
	Regular walk*	5005 (27.6)	2608 (30.2)	2397 (24.5)
	No regular walk	11868 (72.4)	5509 (69.8)	6359 (75.5)

No (%), * *p* < 0.05, NS: non-significant.

**Table 2:** Adjusted^#^ geometric means and 95% CI of blood lead according to classification variables in representative Korean adults.

Classification		All	*p*-Value	Year								Changes in Blood Level Relative to Initial Level.(%)
				2008	2009	2010	2011	2012	2013	2016	2017	
All		1.95 (1.93–1.97)	-	2.37 (2.32–2.42)	2.32 (2.26–2.37)	2.21 (2.17–2.25)	2.12 (2.07–2.17)	1.98 (1.94–2.03)	1.92 (1.88–1.97)	1.74 (1.70–1.78)	1.46 (1.43–1.49)	61.6
Gender	Men	2.13 (2.10–2.15)	**	2.71 (2.65–2.78)	2.64 (2.58–2.71)	2.55 (2.49–2.61)	2.40 (2.34–2.47)	2.23 (2.17–2.28)	2.18 (2.12–2.25)	1.95 (1.89–2.00)	1.65 (1.61–1.69)	60.9
	Women	1.74 (1.72–1.77)		2.00 (1.94–2.06)	1.97 (1.91–2.04)	1.86 (1.81–1.90)	1.82 (1.76–1.88)	1.73 (1.68–1.79)	1.65 (1.60–1.71)	1.51 (1.47–1.55)	1.25 (1.22–1.28)	62.5
Age	<30	1.55 (1.53–1.58)	**	1.94 (1.86–2.02)	1.85 (1.77–1.92)	1.75 (1.68–1.81)	1.66 (1.60–1.72)	1.51 (1.46–1.57)	1.55 (1.47–1.62)	1.34 (1.27–1.42)	1.14 (1.09–1.19)	58.8
	30–39	1.83 (1.80–1.85)		2.24 (2.15–2.32)	2.20 (2.12–2.28)	2.08 (2.01–2.15)	2.01 (1.93–2.10)	1.89 (1.82–1.97)	1.86 (1.80–1.93)	1.62 (1.56–1.68)	1.31 (1.26–1.37)	58.5
	40–49	2.04 (2.01–2.07)		2.49 (2.39–2.59)	2.47 (2.36–2.59)	2.38 (2.28–2.49)	2.23 (2.15–2.32)	2.09 (2.00–2.19)	2.05 (1.97–2.13)	1.82 (1.76–1.89)	1.56 (1.51–1.62)	62.7
	50–59	2.31 (2.28–2.35)		2.78 (2.67–2.89)	2.76 (2.66–2.87)	2.66 (2.57–2.77)	2.57 (2.46–2.69)	2.46 (2.33–2.59)	2.29 (2.20–2.38)	2.13 (2.06–2.21)	1.78 (1.72–1.84)	64.0
	60≤	2.35 (2.31–2.39)		2.65 (2.53–2.77)	2.57 (2.45–2.69)	2.46 (2.34–2.58)	2.44 (2.36–2.53)	2.43 (2.31–2.56)	2.14 (2.04–2.26)	2.13 (2.06–2.20)	1.80 (1.74–1.87)	67.9
Employment	Manual	1.99 (1.97–2.02)	**	2.71 (2.62–2.79)	2.60 (2.52–2.69)	2.46 (2.39–2.53)	2.39 (2.31–2.48)	2.19 (2.11–2.28)	2.14 (2.07–2.21)	1.92 (1.86–1.98)	1.64 (1.59–1.69)	60.5
	Non-manual	1.95 (1.93–1.98)		2.22 (2.12–2.32)	2.26 (2.18–2.34)	2.11 (2.05–2.18)	2.00 (1.93–2.08)	1.95 (1.88–2.02)	1.85 (1.78–1.92)	1.69 (1.64–1.75)	1.33 (1.29–1.37)	59.9
	Others	1.89 (1.87–1.92)		2.18 (2.11–2.24)	2.10 (2.03–2.17)	2.03 (1.97–2.09)	1.92 (1.85–1.98)	1.79 (1.72–1.87)	1.74 (1.67–1.80)	1.60 (1.55–1.65)	1.38 (1.34–1.42)	63.3
Smoking	Non-smoker	1.88 (1.86–1.91)	**	2.06 (2.00–2.12)	2.03 (1.97–2.10)	1.94 (1.89–1.99)	1.89 (1.82–1.95)	1.75 (1.69–1.80)	1.72 (1.67–1.77)	1.54 (1.50–1.58)	1.29 (1.26–1.33)	62.6
	Past smoker	1.94 (1.91–1.97)		2.66 (2.56–2.76)	2.55 (2.44–2.65)	2.47 (2.37–2.58)	2.40 (2.30–2.50)	2.21 (2.11–2.31)	2.12 (2.03–2.22)	1.96 (1.90–2.03)	1.62 (1.56–1.68)	60.9
	Current smoker	2.07 (2.04–2.10)		2.82 (2.72–2.91)	2.75 (2.66–2.84)	2.60 (2.52–2.68)	2.41 (2.33-2.51)	2.33 (2.25–2.41)	2.21 (2.12–2.30)	1.98 (1.90–2.05)	1.69 (1.63–1.75)	59.9
Alcohol	None	1.82 (1.80–1.85)	**	2.20 (2.11–2.29)	2.15 (2.07–2.24)	2.10 (2.01–2.20)	2.03 (1.94–2.12)	1.88 (1.80–1.97)	1.86 (1.78–1.95)	1.67 (1.61–1.72)	1.42 (1.36–1.48)	64.5
	Mild	1.89 (1.87–1.91)		2.23 (2.17–2.29)	2.16 (2.09–2.23)	2.05 (2.00–2.10)	1.98 (1.92–2.04)	1.80 (1.75–1.85)	1.77 (1.72–1.82)	1.61 (1.56–1.66)	1.36 (1.33–1.39)	61.0
	Moderate	2.09 (2.05–2.13)		2.66 (2.53–2.79)	2.66 (2.53–2.79)	2.54 (2.43–2.66)	2.36 (2.21–2.51)	2.28 (2.17–2.40)	2.10 (1.98–2.22)	1.96 (1.86–2.06)	1.59 (1.51–1.68)	59.8
	Heavy	2.21 (2.17–2.25)		2.96 (2.83–3.09)	2.91 (2.79–3.04)	2.66 (2.55–2.78)	2.60 (2.48–2.72)	2.60 (2.47–2.74)	2.48 (2.35–2.63)	2.17 (2.08–2.26)	1.79 (1.71–1.87)	60.5
Residence	Urban	1.94 (1.92–1.95)	*	2.35 (2.29–2.40)	2.29 (2.23–2.34)	2.18 (2.14–2.22)	2.07 (2.03–2.12)	1.96 (1.92–2.01)	1.89 (1.85–1.94)	1.71 (1.67–1.75)	1.43 (1.40–1.46)	60.9
	Rural	1.99 (1.94–2.03)		2.48 (2.39–2.59)	2.46 (2.32–2.61)	2.35 (2.24–2.47)	2.37 (2.19–2.56)	2.08 (1.96–2.20)	2.10 (1.93–2.27)	1.89 (1.80–1.99)	1.59 (1.50–1.69)	64.1
Outdoor activity	Exercise	1.94 (1.92–1.97)	NS	2.41 (2.32–2.50)	2.36 (2.29–2.44)	2.20 (2.13–2.27)	2.11 (2.02–2.21)	1.99 (1.90–2.09)	1.97 (1.88–2.05)	1.71 (1.65–1.77)	1.46 (1.41–1.51)	60.6
	No exercise	1.95 (1.93–1.96)		2.36 (2.30–2.41)	2.31 (2.24–2.37)	2.21 (2.17–2.26)	2.12 (2.07–2.17)	1.98 (1.93–2.03)	1.91 (1.86–1.97)	1.75 (1.71–1.79)	1.46 (1.42–1.49)	61.9
	Regular walk	1.93 (1.91–1.96)	NS	2.30 (2.24–2.37)	2.28 (2.22–2.35)	2.20 (2.13–2.26)	2.08 (2.02–2.14)	1.93 (1.86–1.99)	1.88 (1.82–1.94)	1.70 (1.65–1.76)	1.44 (1.40–1.49)	62.6
	No regular walk	1.95 (1.93–1.97)		2.42 (2.36–2.48)	2.33 (2.27–2.40)	2.21 (2.16–2.26)	2.14 (2.08–2.20)	2.02 (1.96–2.07)	1.95 (1.89–2.01)	1.77 (1.73–1.81)	1.47 (1.44–1.51)	60.7
Menopause (women in their 50s)	Yes (*n* = 1394)	2.08 (2.03–2.13)	**	2.45 (2.29–2.62)	2.42 (2.25–2.60)	2.30 (2.17–2.44)	2.27 (2.15–2.40)	2.26 (2.09–2.43)	2.16 (2.04–2.28)	1.96 (1.86–2.05)	1.57 (1.49–1.65)	75.5
	No (*n* = 370)	1.92 (1.83–2.01)		2.59 (2.37–2.83)	2.56 (2.34–2.79)	2.06 (1.81–2.34)	2.09 (1.80–2.42)	1.87 (1.66–2.10)	1.79 (1.57–2.04)	1.71 (1.56–1.86)	1.36 (1.24–1.50)	70.8

*p < 0.05, ** *p* < 0.01; NS: non-significant; CI = confidence interval; # adjusted for gender, age, region, employment status, smoking & drinking status, residence area, and outdoor activities.

**Table 3:** Adjusted^#^ geometric means and 95% CI of blood cadmium according to classification variables in representative Korean adults.

Classification		All	*p*-Value	Year								Changes in Blood Level Relative to Initial Level.(%)
				2008	2009	2010	2011	2012	2013	2016	2017	
All		0.87 (0.86–0.88)		0.88 (0.85–0.92)	0.91 (0.87–0.95)	0.97 (0.94–1.00)	0.95 (0.92–0.98)	0.91 (0.88–0.94)	0.80 (0.77–0.82)	0.87 (0.84–0.89)	0.72 (0.70–0.74)	81.8
Gender	Men	0.73 (0.72–0.75)	**	0.80 (0.76–0.84)	0.83 (0.79–0.87)	0.88 (0.85–0.91)	0.87 (0.83–0.90)	0.83 (0.80–0.87)	0.74 (0.71–0.77)	0.80 (0.77–0.83)	0.64 (0.62–0.67)	80.0
	Women	1.07 (1.05–1.09)		1.00 (0.96–1.05)	1.01 (0.96–1.06)	1.08 (1.04–1.13)	1.06 (1.02–1.10)	1.02 (0.98–1.06)	0.88 (0.85–0.91)	0.96 (0.92–0.99)	0.83 (0.80–0.86)	83.0
Age	<30	0.57 (0.55–0.58)	**	0.55 (0.51–0.59)	0.57 (0.53–0.62)	0.64 (0.60–0.67)	0.61 (0.57–0.65)	0.57 (0.54–0.61)	0.50 (0.47–0.53)	0.54 (0.50–0.58)	0.42 (0.39–0.45)	76.4
	30–39	0.77 (0.76–0.79)		0.84 (0.78–0.91)	0.83 (0.77–0.89)	0.89 (0.84–0.94)	0.86 (0.81–0.91)	0.82 (0.78–0.87)	0.72 (0.68–0.76)	0.74 (0.70–0.78)	0.60 (0.57–0.64)	71.4
	40–49	1.02 (1.00–1.04)		1.01 (0.96–1.07)	1.06 (1.00–1.12)	1.16 (1.10–1.22)	1.11 (1.06–1.16)	1.09 (1.03–1.16)	0.94 (0.89–0.99)	1.01 (0.96–1.05)	0.89 (0.84–0.94)	88.1
	50–59	1.14 (1.12–1.16)		1.16 (1.09–1.23)	1.23 (1.16–1.30)	1.23 (1.16–1.29)	1.24 (1.17–1.31)	1.20 (1.15–1.25)	1.05 (0.99–1.10)	1.19 (1.14–1.25)	1.00 (0.96–1.05)	86.2
	60≤	1.20 (1.18–1.23)		1.22 (1.15–1.29)	1.22 (1.16–1.29)	1.22 (1.17–1.28)	1.25 (1.19–1.32)	1.24 (1.18–1.30)	1.15 (1.10–1.21)	1.25 (1.21–1.30)	1.08 (1.04–1.13)	88.5
Employment	Manual	0.88 (0.86–0.89)	**	1.02 (0.96–1.09)	1.04 (0.99–1.10)	1.02 (0.98–1.07)	1.05 (1.00–1.11)	1.01 (0.96–1.06)	0.87 (0.83–0.92)	0.94 (0.90–0.98)	0.80 (0.76–0.84)	78.4
	Non-manual	0.85 (0.83–0.87)		0.74 (0.70–0.79)	0.78 (0.72–0.85)	0.91 (0.86–0.96)	0.81 (0.77–0.85)	0.76 (0.72–0.81)	0.71 (0.68–0.75)	0.74 (0.71–0.78)	0.60 (0.57–0.63)	81.1
	Others	0.88 (0.86–0.89)		0.91 (0.86–0.95)	0.93 (0.87–0.98)	0.96 (0.91–1.01)	0.96 (0.92–1.01)	0.93 (0.88–0.98)	0.77 (0.73–0.81)	0.87 (0.83–0.90)	0.73 (0.70–0.76)	80.2
Smoking	Non-smoker	0.75 (0.74–0.76)	**	0.84 (0.81-0.88)	0.86 (0.81–0.90)	0.92 (0.88–0.96)	0.91 (0.88–0.94)	0.89 (0.85–0.93)	0.74 (0.72–0.77)	0.83 (0.80–0.85)	0.67 (0.65–0.69)	79.8
	Past smoker	0.80 (0.78–0.82)		0.76 (0.71–0.81)	0.77 (0.71–0.83)	0.86 (0.81–0.91)	0.78 (0.74–0.82)	0.80 (0.76–0.84)	0.72 (0.67–0.76)	0.78 (0.75–0.82)	0.64 (0.61–0.68)	84.2
	Current smoker	1.21 (1.18–1.24)		1.06 (1.00–1.12)	1.15 (1.09–1.21)	1.15 (1.10–1.21)	1.16 (1.11–1.22)	1.07 (1.02–1.13)	0.98 (0.93–1.03)	1.00 (0.94–1.06)	0.90 (0.85–0.94)	84.9
Alcohol	None	0.86 (0.85–0.88)	**	0.98 (0.93–1.04)	1.03 (0.97–1.10)	1.06 (1.00–1.13)	1.08 (1.02–1.14)	1.00 (0.95–1.06)	0.93 (0.87–0.99)	1.01 (0.97–1.05)	0.85 (0.82–0.90)	86.7
	Mild	0.84 (0.83–0.86)		0.81 (0.78–0.85)	0.84 (0.79–0.89)	0.92 (0.88–0.95)	0.85 (0.82–0.89)	0.86 (0.82–0.89)	0.72 (0.69–0.75)	0.79 (0.76–0.82)	0.65 (0.63–0.67)	80.2
	Moderate	0.89 (0.86–0.91)		0.87 (0.79–0.97)	0.88 (0.81–0.95)	0.95 (0.89–1.01)	0.98 (0.90–1.07)	0.90 (0.83–0.97)	0.84 (0.78–0.92)	0.83 (0.79–0.88)	0.73 (0.68–0.78)	83.9
	Heavy	0.95 (0.93–0.98)		1.01 (0.94–1.09)	1.04 (0.97–1.12)	1.01 (0.94–1.08)	1.08 (1.00–1.16)	1.00 (0.93–1.07)	0.90 (0.84–0.96)	0.97 (0.91–1.03)	0.80 (0.75–0.85)	79.2
Residence	Urban	0.87 (0.86–0.88)	NS	0.86 (0.83–0.90)	0.90 (0.86–0.95)	0.96 (0.93–0.99)	0.92 (0.89–0.95)	0.88 (0.85–0.91)	0.79 (0.76–0.81)	0.85 (0.82–0.88)	0.71 (0.69–0.73)	82.6
	Rural	0.88 (0.85–0.91)		1.00 (0.91–1.09)	0.93 (0.86–1.01)	1.02 (0.94–1.10)	1.09 (1.00–1.20)	1.03 (0.96–1.11)	0.85 (0.80–0.91)	0.96 (0.90–1.02)	0.82 (0.77–0.86)	82.0
Outdoor activity	Exercise	0.85 (0.83–0.86)	*	0.84 (0.79–0.89)	0.86 (0.80–0.92)	0.87 (0.82–0.93)	0.91 (0.85–0.96)	0.87 (0.81–0.94)	0.74 (0.69–0.79)	0.76 (0.72–0.79)	0.63 (0.60–0.66)	75.0
	No exercise	0.88 (0.87–0.89)		0.90 (0.87–0.94)	0.93 (0.89–0.97)	1.01 (0.97–1.04)	0.97 (0.94–1.00)	0.93 (0.90–0.96)	0.82 (0.80–0.85)	0.91 (0.88–0.94)	0.76 (0.74–0.78)	84.4
	Regular walk	0.86 (0.85–0.87)	*	0.81 (0.78–0.85)	0.83 (0.79–0.88)	0.88 (0.85–0.92)	0.89 (0.85–0.93)	0.85 (0.82–0.89)	0.76 (0.73–0.80)	0.83 (0.80–0.86)	0.69 (0.66–0.72)	85.2
	No regular walk	0.88 (0.87–0.89)		0.93 (0.90–0.98)	0.96 (0.92–1.01)	1.02 (0.99–1.06)	0.98 (0.95–1.02)	0.94 (0.91–0.98)	0.82 (0.79–0.85)	0.90 (0.87–0.93)	0.75 (0.73–0.78)	80.6
Menopause (women in their 50s)	Yes (*n* = 1394)	1.29 (1.25–1.32)	**	1.31 (1.19–1.45)	1.36 (1.23–1.50)	1.40 (1.29–1.51)	1.38 (1.28–1.49)	1.45 (1.37–1.54)	1.16 (1.09–1.24)	1.29 (1.22–1.36)	1.09 (1.02–1.17)	83.2
	No (*n* = 370)	1.44 (1.36–1.52)		1.42 (1.28–1.57)	1.53 (1.35–1.72)	1.38 (1.16–1.64)	1.67 (1.32–2.11)	1.51 (1.24–1.84)	1.20 (1.06–1.36)	1.44 (1.26–1.64)	1.38 (1.21–1.56)	97.2

*p < 0.05, ** *p* < 0.01; NS: non-significant; CI = confidence interval; # adjusted for gender, age, region, employment status, smoking and drinking status, residence area, and outdoor activities.

**Table 4:** Differences (95 % CI) in blood lead and cadmium by air concentration of each metal and several classification variables after covariate adjustment# in a subgroup of the metropolitan city population in men and women (KNHANES 2008-2017) (*n* = 7899).

Classification		Men (*n* = 3764)		Women (*n* = 4135)		All	
		Blood Lead	Blood Cadmium	Blood Lead	Blood Cadmium	Blood Lead	Blood Cadmium
Per doubling of air concentration		0.08 (−0.01 to 0.16)	−0.02 (−0.08 to 0.04)	0.10 (0.03 to 0.17)	0.00 (−0.05 to 0.055)	0.09 (0.03 to 0.15)	−0.01 (−0.05 to 0.03)
Gender	Men	-	-	-	-	Reference	Reference
	Women					−0.40 (−0.47 to −0.34)	0.31 (0.28 to 0.35)
Age	<30	Reference	Reference	Reference	Reference	Reference	Reference
	30–39	0.34 (0.24 to 0.45)	0.12 (0.07 to 0.20)	0.15 (0.08 to 0.23)	0.29 (0.24 to 0.34)	0.27 (0.20 to 0.34)	0.20 (0.17 to 0.24)
	40–49	0.66 (0.52 to 0.80)	0.32 (0.26 to 0.38)	0.36 (0.27 to 0.45)	0.66 (0.59 to 0.72)	0.53 (0.45 to 0.61)	0.48 (0.44 to 0.52)
	50–59	0.86 (0.73 to 1.00)	0.47 (0.40 to 0.54)	0.75 (0.65 to 0.84)	0.76 (0.68 to 0.84)	0.82 (0.74 to 0.91)	0.60 (0.552 to 0.65)
	60-	1.04 (0.87 to 1.21)	0.52 (0.45 to 0.58)	0.70 (0.60 to 0.81)	0.89 (0.80 to 0.97)	0.90 (0.79 to 1.01)	0.69 (0.64 to 0.74)
Smoking status	Non-smoker	Reference	Reference	Reference	Reference	Reference	Reference
	Past smoker	0.03 (−0.07 to 0.14)	0.07 (0.02 to 0.11)	0.02 (−0.12 to 0.16)	−0.03 (−0.10 to 0.04)	0.06 (−0.02 to 0.1)	−0.03 (−0.07 to 0.01)
	Current smoker	0.17 (0.06 to 0.27)	0.48 (0.42 to 0.53)	0.11 (−0.03 to 0.26)	0.25 (0.14 to 0.35)	0.18 (0.09 to 0.26)	0.39 (0.35 to 0.44)
Alcohol	None	Reference	Reference	Reference	Reference	Reference	Reference
	Mild	0.06 (−0.05 to 0.17)	−0.02 (−0.07 to 0.04)	0.10 (0.05 to 0.16)	0.01 (−0.04 to 0.06)	0.09 (0.03 to 0.14)	−0.01 (−0.05 to 0.03)
	Moderate	0.39 (0.24 to 0.55)	0.01 (-0.05 to 0.08)	0.34 (0.22 to 0.46)	0.08 (−0.02 to 0.17)	0.39 (0.29 to 0.50)	0.03 (−0.03 to 0.08)
	Heavy	0.50 (0.373 to 0.63)	0.11 (0.05 to 0.18)	0.51 (0.30 to 0.72)	0.14 (0.01 to 0.27)	0.52 (0.42 to 0.62)	0.11 (0.06 to 0.17)

CI = confidence interval; # adjusted for age, region, employment status, smoking and drinking status, residence area, and outdoor activities.
